# The detailed classification of the interlobar artery and the artery crossing intersegmental planes in the right upper lobe

**DOI:** 10.3389/fonc.2023.1195726

**Published:** 2023-05-15

**Authors:** Zhikai Li, Yuhong Kong, Bowen Li, Wenfa Lv, Xiaopeng Zhang

**Affiliations:** ^1^ Graduate School, Hebei Medical University, Shijiazhuang, China; ^2^ Department of Thoracic Surgery, Hebei General Hospital, Shijiazhuang, China; ^3^ Graduate School, North China University of Science and Technology, Tangshan, China; ^4^ Graduate School, Hebei North University, Zhangjiakou, China

**Keywords:** right upper lobe (RUL), anatomy variations, pulmonary artery, anatomical segmentectomy, lobectomy, non-small cell lung carcinoma (NSCLC), video-assisted thoracoscopic surgery (VATS)

## Abstract

**Background:**

With the prevalence of three-dimensional computed tomography bronchography and angiography (3D-CTBA) and the development of anatomical segmentectomy, several studies have analyzed the branching patterns of peripheral segmental arteries in the right upper lobe (RUL). Nevertheless, the detailed classification of the branching patterns of the interlobar artery and the artery crossing intersegmental planes remains unknown. Thus, we conducted a retrospective study to analyze the variations of the interlobar artery and the artery crossing intersegmental planes in the RUL using 3D-CTBA.

**Materials and methods:**

A total of 600 patients with ground-glass opacity (GGO) who had undergone 3D-CTBA preoperatively at Hebei General Hospital between September 2020 and September 2022 were used for the retrospective study. We reviewed the anatomical variations of the RUL arteries in these patients using 3D-CTBA images.

**Results:**

The branching patterns of the RUL artery were classified into the following four categories: trunk superior (Tr. sup), Tr. sup + interlobar artery, Tr. sup + trunk inferior (Tr. inf), and Tr. sup + Tr. inf + interlobar artery. The branching patterns of the interlobar artery were subclassified into four subtypes: posterior ascending artery (A. pos), anterior ascending artery (A. ant), A. pos + A. ant, and ascending artery (A. asc). The artery crossing intersegmental planes contains two types: type A, anterior subsegmental artery crossing intersegmental planes (AX^1^b); type B, recurrent artery crossing intersegmental planes (AX. rec).

**Conclusion:**

The variation types of blood vessels in the RUL are complex. This study explored the detailed classification of the interlobar artery and the artery crossing intersegmental planes. It can help thoracic surgeons understand the anatomy variations, accurately locate lesions before surgery, and effectively plan surgeries.

## 1 Introduction

Lung cancer (LC) is a common malignancy worldwide and is regarded as the leading cause of death (1). The high mortality of LC puts a tremendous strain on public health systems. In recent years, as high-resolution computed tomography (HRCT) is generally applied to health examinations, ground-glass opacity (GGO) is increasingly being confirmed. Surgery remains the best treatment option for early-stage LC. Studies showed that sublobar resection with adequate surgical margins is feasible and effective (2–6), particularly for lesions with a diameter of less than 2 cm and with a consolidation tumor ratio of less than 25%. The main forms of sublobar resection currently include wedge resection and anatomical segmentectomy. Additionally, anatomical segmentectomy preserves better lung function and minimizes lung volume loss. These benefits draw our attention to video-assisted thoracoscopic surgery (VATS) anatomical segmentectomy. However, the presence of anatomical variations may bring great difficulties and challenges to VATS anatomical segmentectomy.

The procedure of performing VATS anatomical segmentectomy is extremely risky with regard to the pulmonary artery as variable pulmonary artery branches are often encountered during dissociation. Without sufficient preoperative anatomy knowledge, it is difficult to accurately mutilate the artery of the target lung segment intraoperatively, which is likely to lead to the conversion of anatomical segmentectomy to lobectomy, which prolongs the surgical time and leads to loss of lung function. Therefore, a comprehensive understanding of the branching patterns of the peripheral segmental arteries is essential for the successful performance of segmentectomy and important to avoid intraoperative pulmonary vessel injury.

In the early days of lung segmental anatomy research, gross anatomical specimens were the primary source of information (7). In recent years, the technology of three-dimensional computed tomography bronchography and angiography (3D-CTBA) is developing rapidly, which extracts high-quality planar image data from computed tomography (CT) scans and creates three-dimensional (3D) virtual models of the lungs, including segments, subsegments, lesions, bronchi, and vessels. Several studies have analyzed the branching patterns of peripheral segmental arteries in the right upper lobe (RUL). However, there is no report showing the classification of the branching patterns of the interlobar artery and the artery crossing intersegmental planes. The aim of this study was to explore the branching patterns of the interlobar artery and the artery crossing intersegmental planes utilizing data from 3D-CTBA. To further advance our understanding of the branching patterns of the pulmonary artery in the RUL, we also compared the results of our study with a similar study that was previously conducted (8).

## 2 Materials and methods

### 2.1 Patient preparation

Between September 2020 and September 2022, 600 patients (336 female and 264 male) who underwent surgeries to treat lesions in the RUL at Hebei General Hospital were enrolled in this study. The mean age was 58 years. All procedures involving human participants in this study were in accordance with the Declaration of Helsinki (revised in 2013). This retrospective study was approved by the Research Ethics Committee at Hebei General Hospital (No. 2022119). The need for patient consent was waived because of the retrospective nature of the study (9).

Inclusion criteria:

1) GGO, with a diameter of less than 2 cm and with a consolidation tumor ratio of less than 25%, located in the RUL;
2) sublobar resection (segmentectomy or wedge resection) was performed; and
3) patients underwent routine chest-enhanced CT examinations preoperatively.

Exclusion criteria:

1) The images presented by enhanced CT lung examination were not clear, which affected the 3D reconstruction of the lung;
2) with a history of right lung surgery; and
3) with a history of pulmonary tuberculosis.

### 2.2 Reconstruction of 3D-CTBA

We performed preoperative chest-enhanced CT using Siemens 64-slice dual-source CT (Somatom Definition) with the contrast agent ioversol 350. A total of 70 ml of contrast medium (ioversol 350) was administered intravenously at a rate of 2–3 ml/s. Contrast-enhanced CT was performed using the fixed-time method. The arterial phase scans were taken 30 s after contrast injection, and the venous phase scans 90 s after contrast injection. The technical parameters used for the Siemens 64-slice dual-source CT were as follows: a collimator thickness of 0.6 mm, a reconstruction layer of 1.25 mm, and an interlayer space of 1 mm (9). By setting a scan start time, the CT values of the pulmonary veins and arteries revealed density variations in the images. The patients were required to hold their breath throughout the CT scan for appropriate bronchial inflation, and precautions were taken to avoid any potential side effects from the contrast agent following the scan. The volume data from both arterial and venous phases were imported into reconstruction software (Infer Operate Thorax Planning), which computed and processed the data before presenting them in 3D-CTBA images (9).

### 2.3 Definition of RUL artery branch

**Figure 1:**
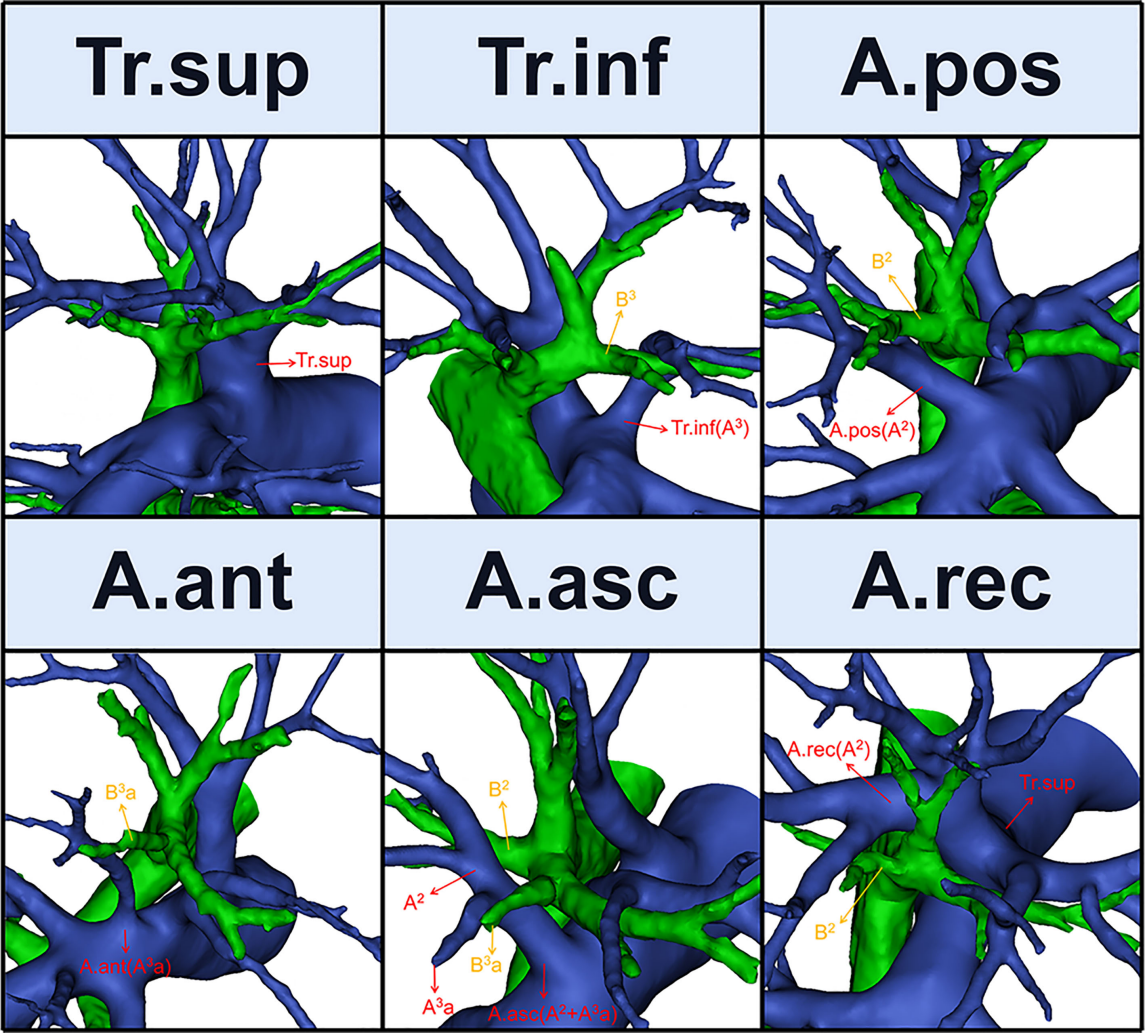
The 3D reconstruction model of Tr. sup, Tr. inf, A. pos, A. ant, A. asc, and A. rec.

**Figure 2:**
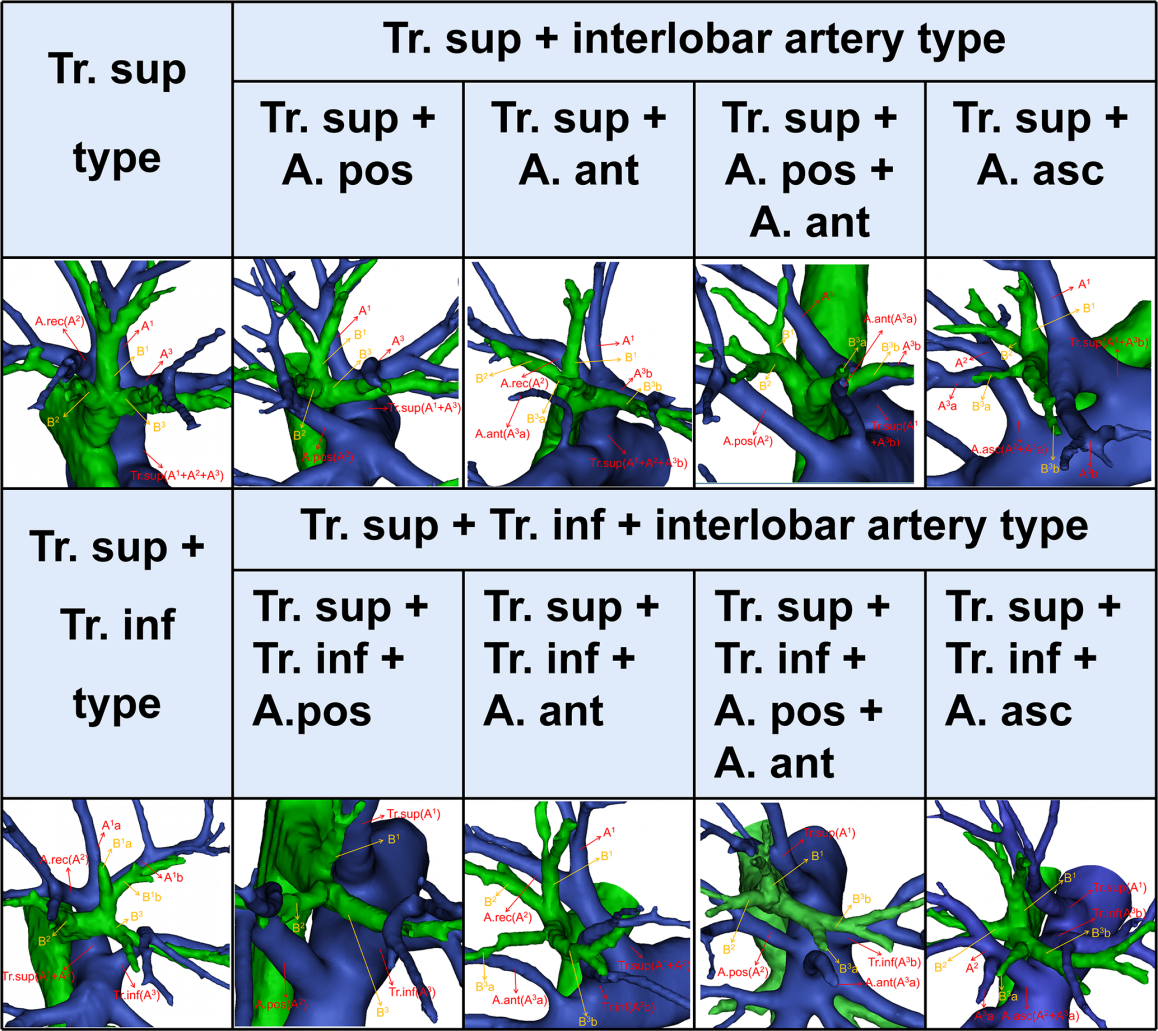
The 3D reconstruction model of the branching patterns of the RUL artery. RUL, right upper lobe.

Six names of RUL artery branches were defined (**Figures 1**, **2**): trunk superior (Tr. sup), trunk inferior (Tr. inf), posterior ascending artery (A. pos), recurrent artery (A. rec), anterior ascending artery (A. ant), and ascending artery (A. asc) (9).

#### 2.3.1 Tr. sup

The Tr. sup is the first branch of the right main pulmonary artery and is often the chief source of the RUL artery (**Figure 1**). Originating from the mediastinal portion of the RUL artery, it lies below the azygos vein arch and flows into the RUL at the anterior side of the RUL bronchus (9).

#### 2.3.2 Tr. inf

The Tr. inf (**Figure 1**), which also originates from the mediastinal portion of the RUL artery and passes anterior to B^3^, has two definitions that depend on whether the Tr. sup is split into upper and lower parts (7–9). If it is, then the Tr. inf is the lower part; if not, it is the second branch of the right pulmonary artery, which arises between the distal region of the Tr. sup and proximal region of the first middle lobe of the pulmonary artery.

#### 2.3.3 A. pos, A. ant, and A. asc

The artery branch originating from the interlobar portion of the right pulmonary artery is located at the posterior side of B^3^ (**Figure 1**). The A. pos is named if it only supplies S^2^, while the A. ant is named if it only supplies S^3^, and the A. asc is named if it supplies both S^2^ and S^3^. Moreover, the A. ant usually arches over the central vein (V. cent) (7, 9).

#### 2.3.4 A. rec

The A. rec is a branch of the Tr. sup and crosses behind B^1^a to supply S^2^ (**Figure 1**) (9).

#### 2.3.5 AX^1^b and AX. rec

**Figure 3:**
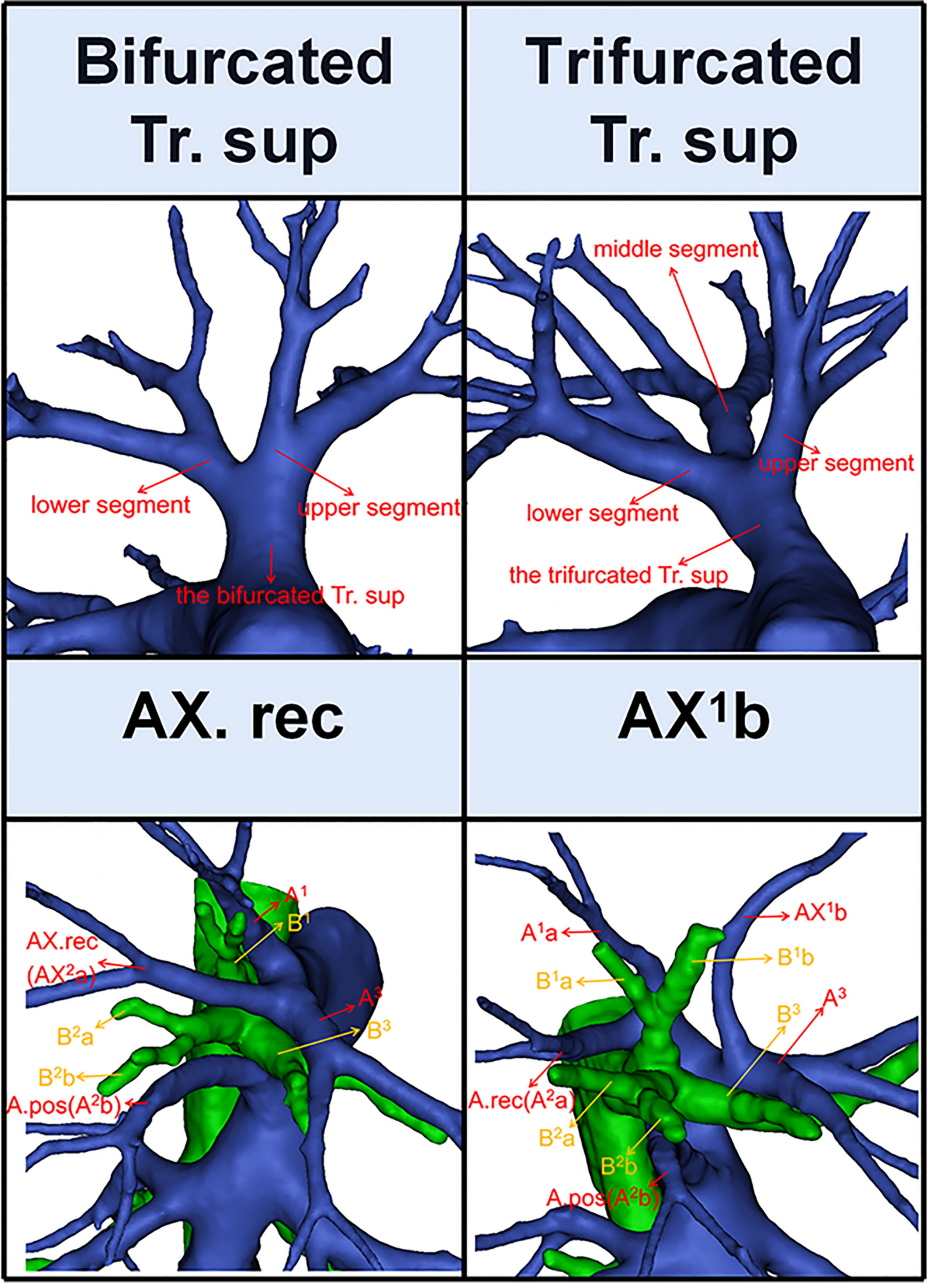
The 3D reconstruction model of the bifurcated Tr. sup, the trifurcated Tr. sup, AX. rec, and AX^1^b.

According to Boyden’s classification principle (7), the Tr. sup was divided into two types. The first type is the bifurcated Tr. sup (**Figure 3**), which is commonly separated into upper and lower segments. The lower segment is principally composed of A^3^. The composition of the upper segment is variable, which is usually composed of either A^1^ and the A. rec or only A^1^. The second type is the trifurcated Tr. sup (**Figure 3**), which is divided into upper, middle, and lower segments. The composition of the upper and lower segments is similar to that of the bifurcated Tr. sup mentioned before. The middle segment is usually composed of the A. rec crossing intersegmental planes (AX. rec).

Thus, A^1^b crossing intersegmental planes (AX^1^b) is named when its origin descends to A^3^ (**Figure 3**); AX. rec is named when it originates from the middle segment of the trifurcated Tr. sup or A^3^ and crosses between B^1^ and B^3^ to supply the S^2^ (**Figure 3**) (9).

### 2.4 Statistics

All statistical analyses were performed using SPSS 23.0 (SPSS, Chicago, IL, USA). Qualitative data were expressed as the number of cases (percentage). Pearson’s chi-square test was used to evaluate the significance of dependencies between the groups. A p-value less than 0.05 was considered statistically significant.

## 3 Results

### 3.1 Branching patterns of RUL artery

The branching patterns of the RUL arteries were classified into four types (**Table 1**; **Figure 2**): type A, Tr. sup (25/600, 4.2%); type B, Tr. sup + interlobar artery (446/600, 74.3%); type C, Tr. sup + Tr. inf (15/600, 2.5%); type D, Tr. sup + Tr. inf + interlobar artery (114/600, 19.0%). According to the supplying range and the number of the interlobar artery branch, four types can be defined: A, A. pos; B, A. ant; C, A. pos + A. ant; D, A. asc. Thus, the “Tr. sup + interlobar artery type” and “Tr. sup + Tr. inf + interlobar artery type” were respectively subclassified into four subtypes (**Table 1**; **Figure 2**). In conclusion, the “Tr. sup + A. pos type” was evident in 272 cases (45.3%) and was the most common type. Moreover, there was no significant difference between the male group and the female group on the branching patterns of the RUL artery (**Table 2**).

**Table 1:** Branching patterns of the right upper lobe artery.

	Our study (n = 600)		Nagashima (n = 263)	
	No.	%	No.	%
Tr. sup type	25	4.2	26	9.9
Tr. sup + interlobar artery type	446	74.3	189	71.9
Tr. sup + A. pos type	272	45.3	NR	–
Tr. sup + A. ant type	16	2.7	NR	–
Tr. sup + A. pos + A. ant type	53	8.8	NR	–
Tr. sup + A. asc type	105	17.5	NR	–
Tr. sup + Tr. inf type	15	2.5	9	3.4
Tr. sup + Tr. inf + interlobar artery type	114	19.0	36	13.7
Tr. sup + Tr. inf + A. pos type	75	12.5	NR	–
Tr. sup + Tr. inf + A. ant type	6	1.0	NR	–
Tr. sup + Tr. inf + A. pos + A. ant type	10	1.7	NR	–
Tr. sup + Tr. inf + A. asc type	23	3.8	NR	–
N/A	–	–	3	1.1

N/A, not available; NR, the type was not referred.

**Table 2:** Distribution of branching types of the right upper lobe artery between male and female patients.

	Male	Female	total	p-Value
Tr. sup type	11	14	25	p > 0.05
Tr. sup + interlobar artery type	202	244	446	
Tr. sup + Tr. inf type	7	8	15	
Tr. sup + Tr. inf + interlobar artery type	44	70	114	
Total	264	336	600	

### 3.2 The anatomical features of A. pos, A. ant, and A. asc

#### 3.2.1 A. pos

**Figure 4:**
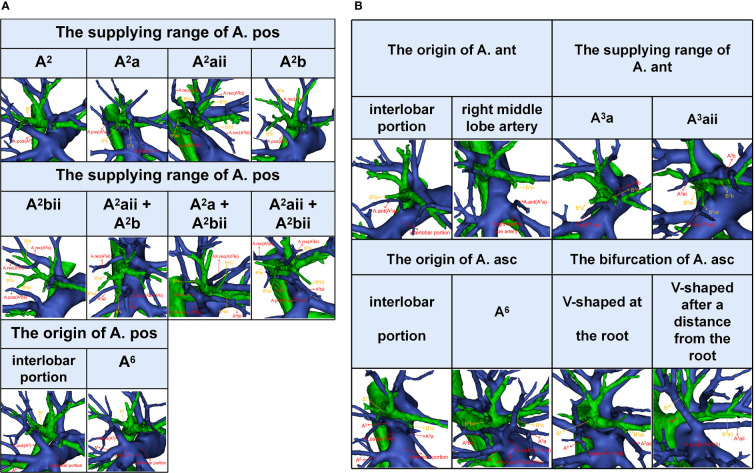
**(A)** The 3D reconstruction model of anatomical features of A. pos. **(B)** The 3D reconstruction model of anatomical features of A. ant and A. asc.

In the following types, “Tr. sup + A. pos”, “Tr. sup + Tr. inf + A. pos”, “Tr. sup + A. pos + A. ant”, and “Tr. sup + Tr. inf + A. pos + A. ant”, the origin of the A. pos has two cases (**Figure 4A**, **Table 3**): A, the interlobar portion (374/410, 91.2%); B, A^6^ (36/410, 8.8%). According to the supplying range of the A. pos, eight categories can be defined (**Figure 4A**, **Table 3**): A, A^2^ (110/410, 26.8%); B, A^2^a (9/410, 2.2%); C, A^2^aii (11/410, 2.7%); D, A^2^b (66/410, 16.1%); E, A^2^bii (55/410, 13.4%); F, A^2^aii + A^2^b (89/410, 21.7%); G, A^2^a + A^2^bii (20/410, 4.9%); H, A^2^aii + A^2^bii (50/410, 12.2%).

**Table 3:** The anatomical features of A. pos.

	Our study (n = 410)	
	No.	%
The origin of A. pos		
Interlobar portion	374	91.2
A^6^	36	8.8
The supplying range of A. pos		
A^2^	110	26.8
A^2^a	9	2.2
A^2^aii	11	2.7
A^2^b	66	16.1
A^2^bii	55	13.4
A^2^aii + A^2^b	89	21.7
A^2^a + A^2^bii	20	4.9
A^2^aii + A^2^bii	50	12.2

#### 3.2.2 A. ant

In the following types, “Tr. sup + A. ant”, “Tr. sup + Tr. inf + A. ant”, “Tr. sup + A. pos + A. ant”, and “Tr. sup + Tr. inf + A. pos + A. ant”, patients can be divided into one of the following two types based on the origins of the A. ant (**Figure 4B**, **Table 4**): A, interlobar portion (78/85, 91.8%); B, right middle lobe (RML) artery (7/85, 8.2%). Types can also be defined according to the supplying range of the A. ant (**Figure 4B**, **Table 4**): A, A^3^a (16/85, 18.8%); B, A^3^aii (69/85, 81.2%).

**Table 4:** The anatomical features of A. ant.

	Our study (n = 85)	
	No.	%
The origin of A. ant		
Interlobar portion	78	91.8
Right middle lobe artery	7	8.2
The supplying range of A. ant		
A^3^a	16	18.8
A^3^aii	69	81.2

#### 3.2.3 A. asc

In the following types, “Tr. sup + A. asc” and “Tr. sup + Tr. inf + A. asc”, the origin of the A. asc was split into two types (**Figure 4B**, **Table 5**): A, interlobar portion (121/128, 94.5%); B, A^6^ (7/128, 5.5%). According to the shape of the A. asc branches, the A. asc can also be classified into two types (**Figure 4B**, **Table 5**): A, the bifurcation of the A. asc is V-shaped at the root (101/128, 78.9%); B, the bifurcation of the A. asc is V-shaped after a distance from the root (27/128, 21.1%).

**Table 5:** The anatomical features of A. asc.

	Our study (n = 128)	
	No.	%
The origin of A. asc		
Interlobar portion	121	94.5
A^6^	7	5.5
The bifurcation of A. asc		
V-shaped at the root	101	78.9
V-shaped after a distance from the root	27	21.1

### 3.3 Branching patterns of RUL segmental arteries

#### 3.3.1 A^1^

**Figure 5:**
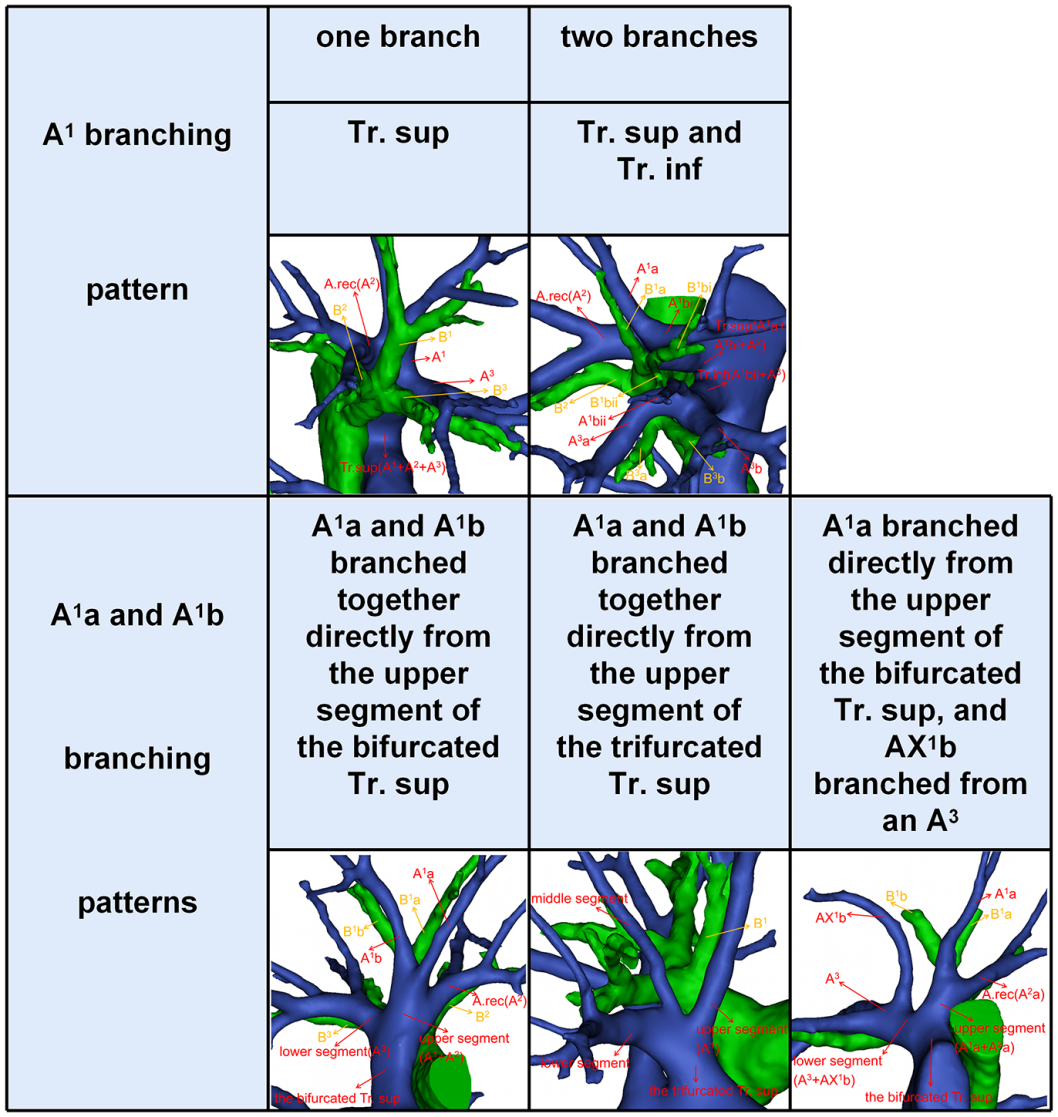
The 3D reconstruction model of the branching patterns of A^1^.

Compared to that of A^2^ and A^3^, the branching pattern of A^1^ shows less diversity. There were two situations in which A^1^ was supplied solely by the Tr. sup in 589 cases (98.2%) while jointly by the Tr. sup and Tr. inf in 11 cases (1.8%), depending on where the A^1^ originated (**Figure 5**, **Table 6**). When A^1^ was supplied solely by the Tr. sup, the branching patterns of A^1^a and A^1^b were divided into two subtypes. In 487 cases (82.7%), the A^1^a and A^1^b branched together directly from the upper segments of the bifurcated Tr. sup or the trifurcated Tr. sup (**Figure 5**). However, in 102 cases (17.3%), only A^1^a branched directly from the upper segments of the bifurcated Tr. sup, and AX^1^b branched from an A^3^ that bifurcated from the Tr. sup (**Figure 5**).

**Table 6:** Branching patterns of A^1^.

	Our study (n = 600)		Nagashima (n = 263)	
	No.	%	No.	%
One branch (Tr. sup)	589	98.2	260	98.9
Two branches (Tr. sup and Tr. inf)	11	1.8	NR	–
N/A	–	–	3	1.1

N/A, not available; NR, the type was not referred.

#### 3.3.2 A^2^

**Figure 6:**
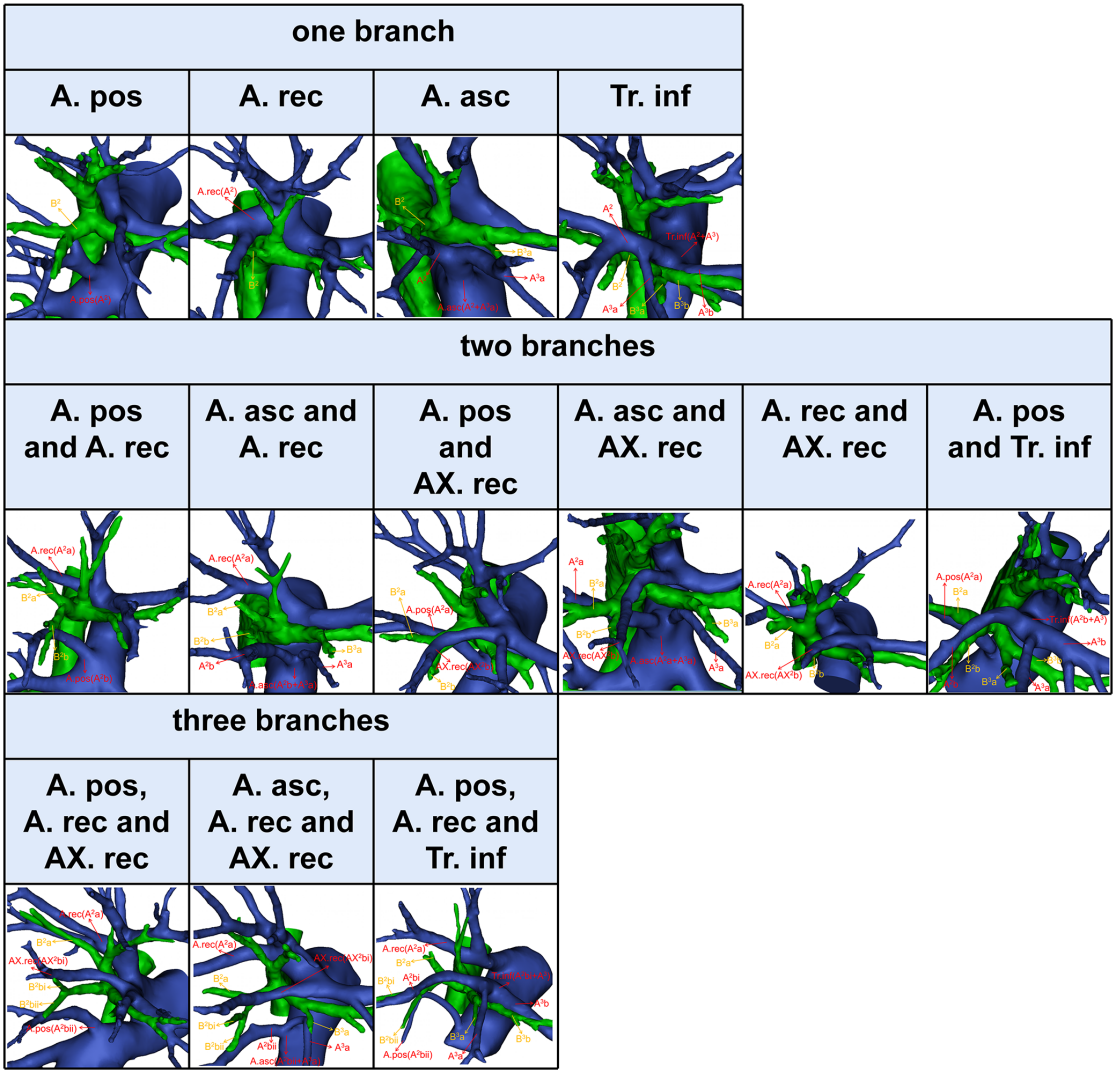
The 3D reconstruction model of the branching patterns of A^2^.

The composition of A^2^ is more complex (9). In this study, the compositions of A^2^ were divided into the following three categories (**Figure 6**, **Table 7**). First, A^2^ is only supplied by one branch of the artery: A. pos (110/600, 18.3%); A. rec (57/600, 9.5%); A. asc (24/600, 4.0%); Tr. inf (2/600, 0.3%). Second, A^2^ is supplied by two branches of the artery: A. pos and A. rec (240/600, 40.0%); A. asc and A. rec (91/600, 15.2%); A. pos and AX. rec (37/600, 6.2%); A. asc and AX. rec (9/600, 1.5%); A. rec and AX. rec (3/600, 0.5%); A. pos and Tr. inf (5/600, 0.8%). Third, A^2^ is supplied by three branches of the artery: A. pos, A. rec, and AX. rec (17/600, 2.8%); A. asc, A. rec, and AX. rec (4/600, 0.7%); A. pos, A. rec, and Tr. inf (1/600, 0.2%). To sum up, the most prevalent forms of A^2^ composition are A. pos and A. rec.

**Table 7:** Branching patterns of A^2^.

	Our study (n = 600)		Nagashima (n = 263)	
	No.	%	No.	%
One branch	193	32.1	123	46.8
A. pos	110	18.3	NR	–
A. rec	57	9.5	39	14.8
A. asc	24	4.0	81	30.8
Tr. inf	2	0.3	1	0.4
AX. rec	NR	–	2	0.8
Two branches	385	64.2	137	52.1
A. pos and A. rec	240	40.0	NR	–
A. asc and A. rec	91	15.2	129	49.0
A. pos and AX. rec	37	6.2	NR	–
A. asc and AX. rec	9	1.5	7	2.7
A. rec and AX. rec	3	0.5	NR	–
A. pos and Tr. inf	5	0.8	NR	–
A. rec and Tr. inf	NR	–	1	0.4
Three branches	22	3.7	NR	–
A. pos, A. rec, and AX. rec	17	2.8	NR	–
A. asc, A. rec and AX. rec	4	0.7	NR	–
A. pos, A. rec, and Tr. inf	1	0.2	NR	–
N/A	–	–	3	1.1

#### 3.3.3 A^3^

**Figure 7:**
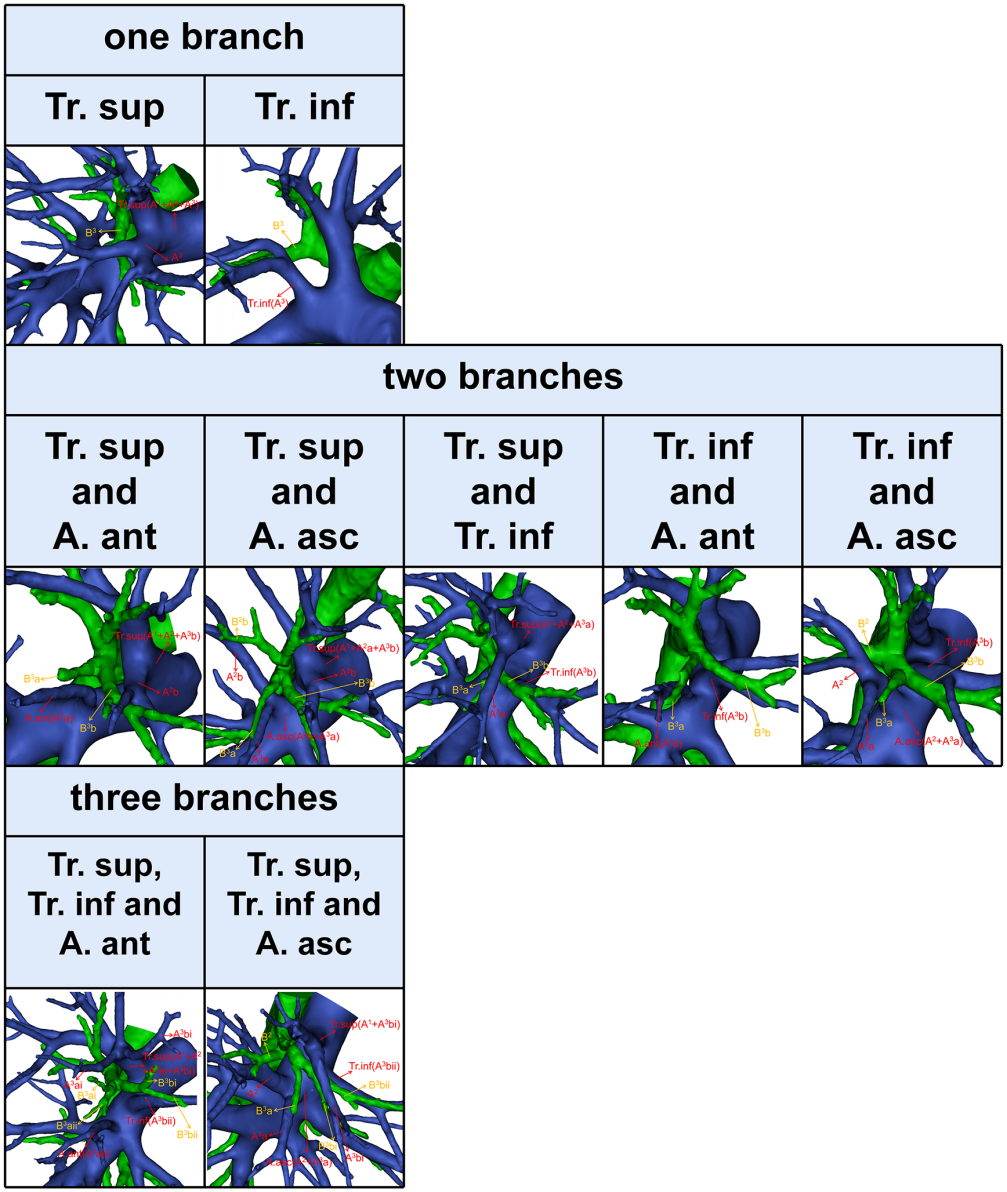
The 3D reconstruction model of the branching patterns of A^3^.

According to the compositions of A^3^, three types can be defined (**Figure 7**, **Table 8**). First, A^3^ is only supplied by one branch of the artery: Tr. sup (297/600, 49.5%) and Tr. inf (42/600, 7.0%). Second, A^3^ is supplied by two branches of the artery: Tr. sup and A. ant (69/600, 11.5%); Tr. sup and A. asc (105/600, 17.5%); Tr. sup and Tr. inf (48/600, 8.0%); Tr. inf and A. ant (9/600, 1.5%); Tr. inf and A. asc (12/600, 2.0%). Third, A^3^ is supplied by three branches of the artery: Tr. sup, Tr. inf, and A. ant (7/600, 1.2%); Tr. sup, Tr. inf, and A. asc (11/600, 1.8%).

**Table 8:** Branching patterns of A^3^.

	Our study (n = 600)		Nagashima (n = 263)	
	No.	%	No.	%
One branch	339	56.5	200	76.1
Tr. sup	297	49.5	180	68.5
Tr. inf	42	7.0	20	7.6
Two branches	243	40.5	60	22.8
Tr. sup and A. ant	69	11.5	NR	–
Tr. sup and A. asc	105	17.5	35	13.3
Tr. sup and Tr. inf	48	8.0	20	7.6
Tr. inf and A. ant	9	1.5	NR	–
Tr. inf and A. asc	12	2.0	5	1.9
Three branches	18	3.0	NR	–
Tr. sup, Tr. inf and A. ant	7	1.2	NR	–
Tr. sup, Tr. inf and A. asc	11	1.8	NR	–
N/A	–	–	3	1.1

N/A, not available; NR, the type was not referred.

## 4 Discussion

With the widespread use of HRCT, increasing numbers of GGO are detected. Some previously published studies have indicated that the prognosis of segmentectomy is no worse than that of lobectomy in patients with early LC (2–6). The anatomical variations of the pulmonary artery make segmentectomy more difficult than lobectomy. Therefore, surgeons must have a comprehensive and accurate understanding of the anatomical characteristics of the branching pattern of the peripheral segmental arteries. Fortunately, advances in the volume-rendering reconstruction technique have enabled the reconstruction of 3D images. 3D-CTBA is a useful tool for thoracic surgeons to identify pulmonary anatomy. An accurate preoperative study can reduce the risk of unexpected bleeding to 2.6% and up to 0 when a printed 3D model is available (10, 11).

However, the branching patterns of the interlobar artery and the artery crossing intersegmental planes are rarely mentioned in previously published reports (7, 8, 12, 13). In the present study, we have comprehensively summarized and classified the branching patterns of the interlobar artery and the artery crossing intersegmental planes using 3D-CTBA.

In the present study (**Table 1**), the “Tr. sup type” was seen in 25 cases (4.2%), which was lower than that reported by Nagashima (9.9%). The rare anatomic variant of the RUL artery was “Tr. sup + Tr. inf type” (2.5%), which was similar to the findings of Nagashima (3.4%). According to the supplying range and the number of the interlobar artery branch, “Tr. sup + interlobar artery” and “Tr. sup + Tr. inf + interlobar artery” can be respectively divided into four subtypes, which have not been reported in previous literature (**Figure 2**, **Table 1**).

As shown in **Figure 2** and **Table 1**, the branching patterns of the RUL artery in this study were somewhat different from those in previous reports (7, 8, 12, 13). The main reason is the specific classification of the interlobar artery as described above. Nagashima defined the interlobar artery as the A. asc. However, the interlobar artery can supply S^2^, S^3^, or S^2^ and S^3^. In this paper, the interlobar artery of the RUL was classified and summarized in detail by 3D-CTBA. The interlobar artery branch was defined as the A. pos when it only supplied S^2^, while the interlobar artery was defined as the A. ant when it only supplied S^3^. Similarly, the interlobar artery branch was nominated as the A. asc if it supplied both S^2^ and S^3^.

Moreover, the definitions of the Tr. inf were ambiguous in the previous studies, so these were redefined in this work. Boyden defined the Tr. inf as the inferior branch of the Tr. sup when it splits into two parts (7). Nagashima defined the Tr. inf as the second branch of the right main pulmonary artery, which arises from the mediastinal portion of the RUL artery between the distal region of the Tr. sup and the proximal region of the first middle lobe of the pulmonary artery (8). However, the two definitions of the Tr. inf are verified in clinical practice (**Figures 1**, **2**). Furthermore, the boundary between the Tr. inf and interlobar artery branch is controversial. Nagashima defined the boundary as the first middle lobe artery (8), while Boyden defined it as B^3^ (7). Boyden indicated that the interlobar artery to the RUL occurs below the level of the highest middle lobe artery at 12% and at the level of 40% in the study of bronchovascular variations of the RML (14). Based on 3D-CTBA data from our study, we verified that the originating location of interlobar artery branches emerges at a higher position than the first middle lobe artery in 69.3% (388/560), below the level in 4.5% (25/560), at the level in 26.3% (147/560). We found that the Tr. inf is located at the anterior side of B^3^ in 97.8% (126/129). Thus, it seems more appropriate to define the boundary as B^3^.

An understanding of the origin of the interlobar artery branch is significant in clinical practice if a safe and accurate lobectomy is to be performed. The anatomical variation, whereby A^6^ shared a common trunk with the A. pos, was found in 36 patients (**Table 3**). A^6^ should be carefully separated from the A. pos before it is cut to avoid damaging A^6^ in the RUL lobectomy (**Figure 4A**). Similarly, the A. pos should be carefully separated from A^6^ before it is divided to avoid injuring the A. pos in the right lower lobe (RLL) lobectomy. Additionally, we also found that the A. asc shared a common trunk with A^6^ in seven cases (**Figure 4B**, **Table 5**). This variation type is rare in clinical practice; however, if it occurs, it causes a great challenge in the performance of lobectomy. Furthermore, a common trunk for the A. ant and RML artery was observed in 8.2% of the cases (**Figure 4B**, **Table 4**). During the RML lobectomy, the A. ant must be protected because a pulmonary artery branch supplied the RUL obstruction that leads to surgical complications such as severe lung edema or extension of the planned lung resection.

The supplying range of the interlobar artery branch has its clinical significance for segmentectomy (**Tables 3**–**5**). For example, for an accurate S^2^b segmentectomy, the intersegmental plane is easily altered without the knowledge of the supply range of the A. pos. If the A. pos supplies S^2^a, a mistaken cut of it will result in an enlarged intersegmental plane (**Figure 4A**). Similarly, if the A. ant supplies S^3^a, excess cutting of the A. ant will result in an enlarged intersegmental plane in S^3^b segmentectomy (**Figure 4B**). If the A. asc supplies a part of S^3^, we need to protect the artery branch supplying S^2^ in the S^3^ segmentectomy (**Figure 4B**). When the bifurcation of the A. asc is V-shaped after a distance from the root, it is necessary to dissect the A. asc in a center-to-periphery direction to identify the bifurcation in S^2^ or S^3^ segmentectomy (**Figure 4B**).

The branching patterns noted in the RUL segmental arteries differed greatly from those of previous reports (**Tables 6**–**8**) (7, 8, 12, 13). This can be explained by the specific classification of the interlobar artery and the artery crossing intersegmental planes. In anatomical segmentectomy, it is significant to understand the branching patterns of pulmonary segmental and subsegmental arteries.

For an accurate S^1^ segmentectomy, it is significant to understand the branching patterns of A^1^ pre-operatively. We found that the branching patterns of A^1^ also had the following branching types: Tr. sup (98.2%), whose incidence is similar to that of Nagashima (98.9%); Tr. sup and Tr. inf (1.8%), which has not been reported in the literature (**Table 6**). When A^1^ branched from the upper segments of the bifurcated Tr. sup or the trifurcated Tr. sup, the Tr. sup must be dissected in a center-to-periphery direction to identify A^1^ (**Figures 3**, **5**). When A^1^bii branching from the Tr. inf runs deep within the lung parenchyma, A^1^bii can be identified after resection of the B^1^ (**Figure 5**). Additionally, we found that AX^1^b bifurcated from A^3^ in 102 cases (**Figure 5**). A mistaken cut at the upper segments of the bifurcated Tr. sup (A^1^a) will result in a narrowed intersegmental plane. Thus, an understanding of the branches and direction of AX^1^b before surgery allows the surgeon to carefully peel off this branch during the intraoperative anatomy, avoiding injury to A^3^.

The branching patterns of A^2^ have significant clinical significance for accurate S^2^ segmentectomy (**Table 7**, **Figure 6**). A^2^ can be composed of the following five components: A. pos, A. asc, A. rec, AX. rec, and Tr. inf (**Figures 1**, **3**, **6**) (9). Moreover, the AX. rec is also a new concept (9). Therefore, three basic approaches to identifying these branches were first reported in our previous paper (9). The A. pos and A. asc can be discriminated by dissecting interlobar fissures (interlobar approach). The Tr. sup can be dissected in a center-to-periphery direction to recognize the A. rec and AX. rec (center-to-periphery approach). The Tr. inf running deep within the lung parenchyma and supplying S^2^ was distinguished after resection of B^2^ (posterobronchial approach). If the A. pos supplies S^2^ (**Figure 6**), the A. pos can be identified by adopting the interlobar approach. If the A. pos and AX. rec supply S^2^, we need to use the interlobar approach and center-to-periphery approach to recognize the A. pos and AX. rec. If A^2^ branched from the A. pos, A. rec, and Tr. inf, the interlobar approach, center-to-periphery approach, and posterobronchial approach should be respectively applied to identify the A. pos, A. rec, and Tr. inf.

Likewise, for an accurate S^3^ segmentectomy, we need to comprehend that S^3^ is supplied by how many arterial branches (**Table 8**, **Figure 7**). If the Tr. sup supplies S^3^, the Tr. sup can be dissected in a center-to-periphery direction to distinguish A^3^. Moreover, when the AX. rec or AX^1^b share a common trunk with A^3^ (**Figure 3**), it is necessary to fully dissociate along the Tr. sup intraoperatively to facilitate the disconnection of A^3^ and the protection of AX. rec, or AX^1^b. When the Tr. inf supplies S^3^, the Tr. inf can be distinguished by dissecting the mediastinal portion of the right main pulmonary artery. If the A. ant supplies S^3^a and the Tr. sup supplies A^3^b (**Figure 7**), the A^3^a can be identified by dissecting interlobar fissures, and the Tr. sup should be dissected in a center-to-periphery direction to identify A^3^b. Therefore, it is significant to conduct a comprehensive and thoughtful investigation of anatomical variations preoperatively.

The incorrect vascular identification may lead to surgical complications in segmentectomy. Surgical procedure changes according to vascular variations, and therefore, accurate preoperative recognition of variations is a mainstay when planning RUL segmentectomy. Therefore, preoperative 3D-CTBA to understand the branching patterns of the segmental arteries in the RUL is necessary to perform an accurate segmentectomy and subsegmentectomy.

## 5 Conclusions

This is the first report to explore the detailed classification of the interlobar artery and the artery crossing intersegmental planes. We believe that our pulmonary artery data and our new nomenclature will facilitate preoperative simulation and intraoperative navigation when RUL segmentectomy is planned and performed.

## Data availability statement

The raw data supporting the conclusions of this article will be made available by the authors, without undue reservation.

## Ethics statement

This retrospective study was approved by the Research Ethics Committee at Hebei General Hospital (No. 2022119). The need for patient consent was waived because of the retrospective nature of the study.

## Author contributions

ZL: project design and initiation, data analysis, and manuscript writing. YK: project design and initiation, data analysis, and manuscript writing. BL: project design and initiation, data analysis, and manuscript writing. WL: data collection. XZ: supervisor. All authors contributed to the article and approved the submitted version.
